# Application of dural suturing in the endoscopic endonasal approach to the sellar region

**DOI:** 10.3389/fsurg.2022.944663

**Published:** 2022-08-18

**Authors:** Zhiyuan Liu, Liang Zhao, Yu Wang, Kexiang Dai, Ailin Lu, Peng Zhao

**Affiliations:** Department of Neurosurgery, First Affiliated Hospital of Nanjing Medical University, Nanjing, China

**Keywords:** endoscopic endonasal approach, dural suture, sellar region, cranial base, pituitary tumor

## Abstract

**Objectives:**

The endoscopic endonasal approach (EEA) is widely used in the treatment of cranial base tumors. Skull base reconstruction is a crucial part of EEA, which has a great impact on patients’ prognosis. In this study, we report our experience with sellar dural suturing in cranial base reconstruction and retrospectively analyze its effect.

**Methods:**

The clinical data of 134 patients who suffered intraoperative CSF leakage and underwent EEA surgery in the Department of Neurosurgery of the First Affiliated Hospital of Nanjing Medical University from October 2018 to November 2020 were retrospectively collected and analyzed. According to whether sellar dural suturing was performed during the operation, they were divided into a suture group (55 cases) and a control group (79 cases).

**Results:**

The results showed that dural suturing of the sellar floor effectively reduced the postoperative hospitalization duration (*p* = 0.026) and the use rates of lumbar drainage (*p* = 0.047), autologous fat transplantation (*p* = 0.038), and pedicled nasoseptal flaps (*p* = 0.026).

**Conclusion:**

Sellar dural suturing under endoscopy is a promising and effective method for cranial base reconstruction in EEA surgery and is worthy of clinical application.

## Introduction

The complex anatomical structure of the sellar region leads to a high incidence of a series of tumors in this region, accounting for approximately 10%–15% of intracranial tumors and mainly including pituitary adenomas, Rathke cleft cysts, craniopharyngiomas, meningiomas, chordomas, and metastases (1). In the last two decades, significant advancements have been made in the field of cranial base surgery. The surgical approach to cranial base intradural lesions has largely progressed through three stages, i.e., craniotomy, the microscopic endonasal approach (MEA), and the endoscopic endonasal approach (EEA). Currently, EEA is the mainstream surgical method applied to most sellar region lesions (2). EEA does have incomparable advantages over traditional craniotomy and MEA (3, 4). However, postoperative cerebrospinal fluid (poCSF) leakage has remained a problem for neurosurgeons and patients. poCSF leakage prolongs the hospitalization of patients, increases the costs incurred by patients, and may also lead to a series of complications, such as infection, headache from low intracranial pressure, subdural hemorrhage, and pneumocranium. Furthermore, mortality due to infection caused by CSF leakage is not rare (5–7). With the progress that has been made in cranial base reconstruction and the development of reconstruction materials, the incidence of poCSF has decreased significantly. Reconstruction methods include autologous fat transplantation and tamponade, abdominal or thigh fascia transplantation, and pedicled nasoseptal flap (NSF) repair, among others. Although these repair materials are self-derived, easy to obtain, and carry no risk of rejection, they may cause other complications, such as ectopic fat compression, long-term pain at the fascia acquisition site, decreased ability for nasal self-purification, long-term empty nose syndrome (ENS), and decreased sense of smell (8–10). In recent years, sellar dural suturing has been gradually applied for cranial base reconstruction in EEA surgery. This method can provide solid sellar support without any autologous or allogeneic transplantation materials only if the sutures are firm, and this technique is challenging for the operator. Since October 2018, the First Affiliated Hospital of Nanjing Medical University has made a preliminary attempt to perform sellar dural suturing in patients with CSF leakage during EEA surgery. Here, we summarize our experience with sellar dural suturing and explore its value in EEA surgery through retrospective analysis.

## Materials and methods

### Patients

We retrospectively analyzed the cases of 134 patients who suffered intraoperative CSF leakage during EEA surgery in the Department of Neurosurgery of the First Affiliated Hospital of Nanjing Medical University from October 2018 to November 2020. All these operations were performed by three cranial base surgical teams with at least one senior expert. Among them, the team of Professor Zhao performed dural suturing in all patients with intraoperative CSF leakage for a total of 55 patients. The other two cranial base surgery teams did not perform this procedure on any patients. Patients who were not operated on by a surgical team with at least one senior expert (116 cases) or who did not suffer intraoperative CSF leakage (307 cases) were excluded. Relevant data were collected and analyzed. Intraoperative CSF leaks were classified by Kelly grade (11): Grade 0, no leak observed (these cases were not included in the study); Grade 1, small leak without obvious diaphragmatic defect; Grade 2, moderate leak; and Grade 3, large diaphragmatic/dural defect. This retrospective study was approved by the ethics committee, and consent was obtained from all patients.

### Operative techniques

**Figure 1:**
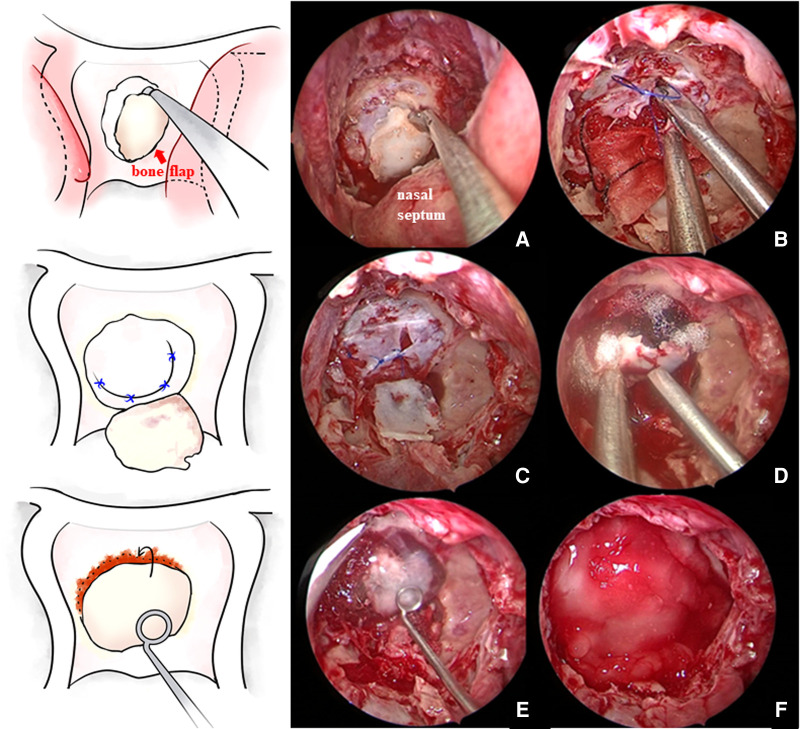
(**A**) *In situ* bone flap was reserved when opening the sellar floor. (**B,C**) After tumor resection and complete hemostasis, the sellar floor dura mater was directly sutured in patients without a sellar floor dura mater defect. (**D**) Then, the bone flap was restored. (**E,F**) Fibrin glue was applied and then covered with artificial dura mater.

Each patient was positioned in a supine position with the head tilted back 5–10° and turned to the right 15° under general anesthesia with endotracheal intubation. All surgeries were performed with the two-surgeon, binostril, and four-hand technique. The right nasal cavity was routinely chosen as the operation channel, with the left side as the auxiliary channel. The nasal septal mucosa was cut and pushed to the right side. Then, the anterior wall of the sphenoid sinus and the sellar base floor were opened under the mucosa, and the sellar base bone flap was retained *in situ* as much as possible (Figure 1A). Before cutting the dura mater, sufficient hemostasis of dural blood vessels should be achieved with bipolar electrocoagulation. After incising the dura mater, additional electrocoagulation of the dura needs to be avoided to prevent dural contracture, which could increase the difficulty of suturing. When cutting the dura mater in a “U” shape, it was necessary to remember to reserve the suture position and ensure that the bone window edge was at least 2 mm away from the dural window edge to facilitate needle entry. Then, the normal pituitary tissue was identified carefully, with reference to preoperative images, and the tumor was resected in blocks. For tumor tissue inside the parasellar cavernous sinus or surrounding the internal carotid artery, the course of blood vessels and the distance between the tumor and blood vessels were detected by transcranial Doppler (TCD), and the tumor was removed with surgical instruments of a certain curvature using an angled endoscope.

**Figure 2:**
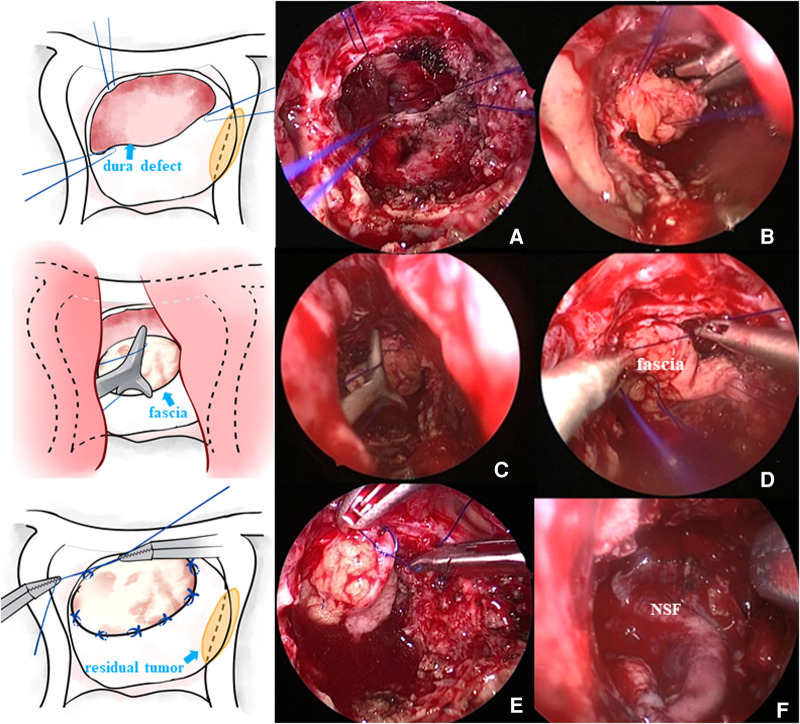
(**A**) Needle was inserted at the edge of the defect dura mater, and then the fascia graft was sutured outside the nasal cavity. (**B**) Fascia graft was pressed into the sellar floor. (**C**) Knot was pushed outside the nasal cavity into the operating area. (**D**) Knot was tightened. (**E**) Fascia graft was further sutured and fixed in the nasal cavity. (**F**) Some patients needed pedicled NSF placement to further prevent poCSF leakage.

After tumor resection and hemostasis, the surgeons began the skull base reconstruction. The same meticulous multilayered reconstructions were applied in two groups. The procedures of skull base reconstruction in the control cohort were the same as those in the suture cohort, except for the dural suturing. The steps of skull base reconstruction for a typical Grade 3 intraoperative CSF leak are as follows. First, the sellar diaphragm fistula was repaired with autologous fascia or fat. At the same time, artificial meninges were applied to the sellar diaphragm to provide additional support. Then, a layer of fibrin glue was usually applied on and beside the artificial meninges. Afterward, the sellar region was filled with gelatin sponge material or autologous fat, depending on the size of the cavity. After that, a layer of artificial meninges was placed inside the dura. Then, the sellar base dura mater was sutured with approximately 4–6 stitches, depending on the length of the dural opening, of 5–0 suture material (Figure 1). Due to the narrow operating space of the nasal cavity and the use of long, gun-like instruments, suturing during the operation is difficult, and adopting an operating mode consisting of two people and four hands is recommended. The assistant operates the endoscope, while the operator holds the special needle holder in the right hand and the suction device (or another needle holder) in the left hand. Usually, application of the Duncan slip knot (12), that is, tying a knot outside the nasal cavity and then pulling it into the sphenoid sinus operation area for tension and reinforcement, is recommended (Figure 2). If there were obvious defects in the sellar floor dura, autologous fascia could be used for suturing repair (Figure 2). After suturing, the dura was covered with a thin layer of hemostatic gauze and fibrin glue. The sellar floor was reset with the *in situ* bone flap (Figure 1) and covered with artificial dura (Figure 1). Most patients with Grade 3 leakage underwent pedicled NSF repair (Figure 2), which is not used routinely in patients with Grade 1 or 2 leakage. Preventive lumbar drainage (LD) was not conducted routinely in either group. Only three patients in the control group underwent preventive LD, and none of the patients in the suture group underwent preventive LD. Finally, the sphenoid sinus was filled with gelatin sponge material for support, and one or both nasal cavities were filled with a polyvinyl fluoride (PVF) expansion sponge. The main procedures of skull base reconstruction for Grade 1 or 2 intraoperative CSF leak were roughly the same as those for Grade 3, except that one or several steps were not required in some cases. For example, for patients without sellar diaphragm fistula, repairs of sellar diaphragm defects were not needed; NSF repair may not be necessary for a certain number of patients with Grade 1 or 2 intraoperative CSF leak; and autologous fat filling was unnecessary if the tumor cavity was not large. Certainly, solid skull base reconstructions are necessary for preventing poCSF leakage, but surgeons should still probably be cautious about invasive procedures used for the aim of skull base reconstruction. Some patients may complain about the prolonged discomfort of the nasal cavity after harvesting NSF or the pain and scar at the fascia acquisition site. In our institution, a Valsalva maneuver was routinely performed by the anesthesiologist to help surgeons determine whether the procedures of skull base reconstruction were sufficient and complete. Experienced senior surgeons would determine that certain invasive repair techniques were no longer needed for certain patients on the basis of their experience, the degree of intraoperative CSF leak, and the results of the vasalva maneuver.

### Postoperative management

All patients remained in the supine position for 24 h after the operation. After 24 h, the head of the bed could be properly raised to no more than 30°, but they were not allowed to ambulate within 72 h. In the first 24 h after the operation, parenteral nutrition replaced oral ingestion. After that, patients were allowed to consume soft high-protein food as appropriate. The patients were instructed to avoid any situations that may lead to an increase in intracranial pressure, such as coughing, sneezing, and straining during defecation, and glycerin enema lotion was provided at the head of the bed. The patients and their families were asked to pay close attention to whether there was clear fluid outflow from the nasal cavity or throat. If such outflow was observed, the patients and their families were asked to report it to the bed nurse or doctor in a timely manner to confirm whether there was poCSF leakage. If poCSF leakage was confirmed, the patients continued to stay in a supine position or underwent LD; if there was no poCSF leakage, patients were allowed to ambulate, use the bathroom or eat food briefly after 72 h. A few patients with a high risk of poCSF leakage were treated with preventive LD; these patients were mainly in the control group. The PVF expansion sponge was removed from the operative and auxiliary sides on the second and third day, respectively, after the operation. If there were no special complications, the patients were discharged on the fifth or sixth day after the operation. For patients with poCSF leakage, if LD and absolute bed rest for 4–5 days were ineffective, surgical repair was carried out. In these cases, we used the EEA on the sphenoid sinus. Then, we removed necrotic tissue and searched carefully for the fistula. Subsequently, we obtained autologous fascia and minced muscle from the thigh, covered the fistula with thigh fascia, and spread minced muscle around the fascia. In our experience, the scar tissue formed by the minced muscle is helpful for preventing poCSF rhinorrhea. Finally, we filled the sphenoid sinus with autologous fat or hemostatic sponge material.

### Outcome measures

Regarding perioperative measures, the intraoperative blood loss, total tumor resection rate, dural suturing duration, incidence of poCSF leakage, application of autologous grafts, application of LD, postoperative hospitalization duration, and incidence of infection and other complications were recorded. Regarding prognostic measures, the patients were followed up by telephone at 1 and 6 months after the operation and regular outpatient reexaminations at 3 months and 1 year after the operation. The follow-up measures included the incidence of recurrence, which was evaluated by pituitary hormone serological examination, cranial magnetic resonance imaging (MRI), and visual field examination, among other methods, as well as the incidence of delayed poCSF leakage and nasal complications.

### Statistical analysis

SPSS 26.0 statistical software was used for statistical analysis. The *t*-test was used for measurement data, the chi-square test was used for count data (Fisher's exact test was used when the expected event number was less than five), and the rank-sum test was used for grade data. Multivariate analyses were performed to confirm the significance observed. *p* < 0.05 was considered to indicate statistical significance.

## Results

### General characteristics

A total of 134 patients were included in this study, with 55 patients in the suture group and the other 79 patients in the no-suture (control) group. The risk factors for poCSF include age, maximum diameter of tumor on imaging, sex, recurrent surgery (surgery after tumor recurrence), degree of intraoperative CSF leakage, extended EEA surgery, and tumor pathology (11, 13–17). These factors were compared between the suture and control groups (Table 1). Statistical results confirmed no difference between these two groups in terms of age (*p* = 0.417), sex (*p* = 0.320), tumor diameter (*p* = 0.523), proportion of recurrent surgery (*p* = 0.668), proportion of extended EEA surgery (*p* = 0.700), and degree of intraoperative CSF leak (*p* = 0.986).

**Table 1:** General characteristics.

Variables	Group		*p*-value
	Suture (*n* = 55)	Control (*n* = 79)	
Age	49.600 ± 15.522	47.443 ± 14.564	0.417
Maximum diameter on imaging	2.935 ± 0.865	3.035 ± 0.886	0.523
Sex (male)	21	36	0.320
Recurrent cases	5	9	0.668
Kelly grade (11)			0.986
1	32	46	
2	17	24	
3	6	9	
High-risk pathologies	10	20	0.330
Extended EEA	11	18	0.700

The postoperative pathological results revealed 98 (suture group/control group: 42/56) cases of pituitary adenoma, 12 (5/7) cases of craniopharyngioma, 11 (3/8) cases of meningioma, 4 (2/2) cases of chordoma, 6 (3/3) cases of Rathke cysts, 1 (0/1) case of renal clear cell carcinoma metastasis, 1 (0/1) case of lymphoma, and 1 (0/1) case of nasopharyngeal carcinoma (misdiagnosed as chordoma before the operation). According to previous clinical studies and literature reports (16), the incidence of poCSF leakage is higher in cases of malignant tumors, craniopharyngiomas, meningiomas, and chordomas than in cases of pituitary adenomas and Rathke cysts. Therefore, these four types of cases were considered high-risk cases; there were 10 high-risk cases in the suture group and 20 high-risk cases in the control group. However, there was no significant difference in the pathological composition of the two groups, as determined by the chi-square test (*χ*^2^ = 0.950, *p* = 0.330).

In summary, there was no significant difference between the suture group and the control group in age, sex, maximum tumor diameter on imaging, rate of recurrent surgery, grade of intraoperative CSF leakage, or pathological category. The surgical effect indicators for the suturing and control groups were comparable.

### Comparison of surgical effect with and without intraoperative dural suturing

In the suture group, the average operative duration was 144.109 ± 52.018 min, the average intraoperative blood loss was 161.455 ± 108.685 ml, and the average postoperative hospital stay (not including the operation day or discharge day) was 5.800 ± 1.623 days. In the control group, the average operative duration was 135.076 ± 56.773 min, the average intraoperative blood loss was 170.848 ± 76.380 ml, and the average postoperative hospital stay was 7.063 ± 3.911 days. In the suture group, no patients required preventive LD, and poCSF leakage occurred in one patient and healed after LD. There were no cases of infection. Twenty-two patients underwent autologous fat transplantation, 11 patients underwent autologous fascia transplantation, and 6 patients underwent pedicled NSF repair. Complete resection was achieved during the operation in 50 cases, and reoperations were required in 5 cases due to tumor recurrence during the follow-up period. In the control group, nine patients required LD (preventive in three cases and therapeutic in six cases), and poCSF leakage occurred in six patients; of these six patients, five patients underwent six repair surgeries and one patient was healed after LD. Three patients developed a postoperative infection, 46 patients underwent autologous fat transplantation, 25 patients underwent autologous fascia transplantation, and 21 patients underwent pedicled NSF repair. Total resection was achieved in 69 cases, and reoperations were performed in 7 cases due to tumor recurrence. These data were statistically analyzed; measurement data were tested by the independent-sample *t*-test, and count data were tested by the Pearson chi-square test or Fisher exact test (Table 2). It was proved that the suturing group has lower use rates of LD (*p* = 0.047), autologous fat (*p* = 0.038), and NSF (*p* = 0.026). The postoperative hospitalization time of the suturing group was significantly shorter than that of the control group (*p* = 0.026). There was no significant difference in the incidence of poCSF leakage between the two groups (1.818% vs. 7.595%, *p* = 0.239), which we believe a larger sample is needed for further validation.

**Table 2:** Comparison of surgical effect with and without dural suturing.

Variables	Group		*p*-value
	Suture (*n* = 55)	Control (*n* = 79)	
Operative duration (min)	144.109 ± 52.018	135.076 ± 56.773	0.354
Intraoperative blood loss (ml)	161.455 ± 108.685	170.848 ± 76.380	0.561
Postoperative hospitalization (day)	5.800 ± 1.623	7.063 ± 3.911	0.026
poCSF leakage	1	6	0.239
LD	1	9	0.047
Infection	0	3	0.269
Autologous fat transplantation	22	46	0.038
Autologous fascia transplantation	11	25	0.135
NSF repair	6	21	0.026
Complete resection	50	69	0.519
Repair operation	0	5	0.078
Surgery for tumor recurrence	5	7	0.963

### Multivariate analysis of postoperative hospitalization and invasive operations

It has been statistically proved that the patients in the suturing groups have shorter postoperative hospitalization and lower utilization rates of some invasive operations (LD, NSF, and autologous fat) than those in the control group. In literature (11, 15–18), it is well known that extended EEA surgeries and surgeries for recurrent lesions need more solid skull base reconstructions. In these cases, more invasive repairing methods may be applied and patients may have a longer postoperative hospitalization duration. These factors may have an impact on the significance observed by the authors, so multivariate logistic regression and linear regression were performed to confirm the difference made by dural suturing between the two groups. The results are shown in Table 3. Multivariate regression showed that dural suturing was the independent protective factor for short postoperative hospitalization (*p* = 0.040, *β* −0.174, CI 95% −2.236–0.055), low use rate of NSF (*p* = 0.029, OR 3.532, CI 95% 1.140–10.945), and autologous fat (*p* = 0.018, OR 2.616, CI 95% 1.179–5.808). That means sellar dural suturing does bring certain benefits to patients regardless of age, gender, or surgery type (extended EEA or not, recurrent surgery or not).

**Table 3:** Regression analysis in risk factors of the observed significances.

Variables	Postoperative hospitalization		LD		NSF		Autologous fat	
	*p*-value	*β* (CI 95%)	*p*-value	OR (CI 95%)	*p*-value	OR (CI 95%)	*p*-value	OR (CI 95%)
Age	0.200	−0.110 (−0.060–0.013)	0.081	0.960 (0.917–1.005)	0.205	1.024 (0.987–1.061)	0.051	1.027 (1.000–1.055)
Sex	0.911	0.010 (−1.071–1.200)	0.330	0.481 (0.110–2.101)	0.207	0.521 (0.190–1.433)	0.416	1.404 (0.620–3.176)
Dural suturing	0.040	−0.174 (−2.236–0.055)	0.086	6.434 (0.770–53.782)	0.029	3.532 (1.140–10.945)	0.018	2.616 (1.179–5.808)
Extended EEA	0.003	0.265 (0.746–3.451)	0.246	0.411 (0.091–1.845)	0.002	0.188 (0.066–0.535)	0.000	0.089 (0.026–0.302)
Recurrent surgery	0.845	0.016 (−1.571–1.916)	0.997	0.996 (0.109–9.093)	0.003	0.141 (0.039–0.512)	0.064	3.761 (0.925–15.292)

### Suture duration analysis

**Figure 3:**
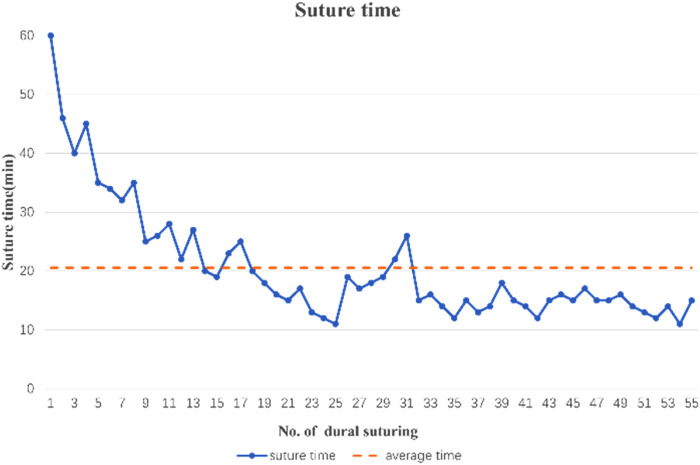
Time required for dural closure. The dotted line represents the average time.

Since all the operations in the suture group were completed by the same surgical team, the patients in the suture group could be divided into three groups according to the surgery dates. The first 20 operations were designated group A, with an average suture duration of 29.800 ± 10.961 min; the middle 20 operations were designated group B, with an average suture duration of 16.050 ± 3.542 min; and the last 15 operations were designated group C, with an average suture duration of 14.267 ± 1.611 min (Figure 3). These three groups of data were statistically analyzed, and the Kruskal–Wallis *H* test was used. A significant difference in the suture duration was found between groups A and B (*p* = 0.001) or C (*p* = 0.001) but not between groups B and C (*p* = 0.176). These results suggest that although performing sellar dural suturing is initially difficult and requires a good deal of time, the learning curve for this technique is steep for senior neurosurgeons, and it can be mastered after a limited number of practices, allowing the timing of the operation to be controlled within an acceptable range.

## Discussion

The widespread application of neuroendoscopy has promoted the development of cranial base surgery into a new era. The anatomical structure of the cranial base is complex, with numerous neurovascular intersections; this area used to be a restricted zone in neurosurgery. During a traditional craniotomy, neurosurgeons need to retract brain tissue to expose lesions and remove tumors through a space between nerves and vessels, and it is difficult to directly observe the dorsal structure of tumors in this region. Therefore, injuries caused by pushing or pulling may be inevitable and suprasellar extended lesions may not be easily removed completely. The application of neuroendoscopy addressed these problems. EEA surgery takes advantage of a natural lumen to directly reach the ventral or basal part of such lesions, allowing neurosurgeons to cut off the tumor base and blood supply early. In addition, neuroendoscopy has its own unique advantages compared with microscopy, including a wider viewing angle, a deeper surgical field, and less trauma. However, the widespread application of neuroendoscopy in cranial base surgery, especially in cases of highly invasive lesions such as chordomas and craniopharyngiomas, also presents higher requirements for cranial base reconstruction.

Cranial base reconstruction has always been a research hotspot in cranial base surgery. On the one hand, the purpose of cranial base reconstruction is to provide rigid support for cranial base tissue and prevent brain tissue from herniating *via* the surgical channel. On the other hand, it is performed to close the skull cavity and build a physical barrier inside and outside the skull to prevent CSF leakage or retrograde infection. The basic principle of cranial base reconstruction is meticulous multilayer reconstruction. With the continuous improvement of reconstruction technology and repair materials, the incidence of poCSF leakage has been greatly reduced. At present, the commonly used cranial base reconstruction methods can effectively reduce the incidence of poCSF leakage. However, the materials used in these methods may also have some adverse effects, as follows. (a) Pedicled NSF: the ability for self-purification of the nasal cavity decreases after the operation, resulting in ENS and anosmia, which can seriously affect the quality of life (19, 20); if the pedicled nasal mucosal flap is necrotic, it can become a source of infection and cause intracranial infection (21). (b) Thigh or abdominal fascia: long-term pain at the fascia acquisition site after the operation is the main sequelae; in addition, the scar caused by fascia acquisition may cause embarrassment to some patients. (c) Fat transplantation: fat displacement may occur after the operation; although the incidence rate is low, repair failure or ectopic compression symptoms may occur. (d) Preventive LD: as an invasive operation, LD may lead to secondary infection, and these patients are required to always remain in a supine position after the operation; this mandatory posture not only brings many inconveniences to daily life but is also very painful. Under these circumstances, our cranial base surgical team made a preliminary attempt to apply sellar dural suturing in patients with intraoperative CSF leakage during the EEA operation.

Sellar dural suturing can minimize dural defects, reconstruct the cranial base in a more natural way, and reduce the application of autologous or artificial materials to effectively avoid the disadvantages of the above repair methods. In 2004, Megyesi et al. (22) confirmed *in vitro* that simple intermittent suturing of the dura mater has a significant effect on preventing CSF leakage. From 2006 to 2009, Nishioka et al. (23) performed direct dural suturing in 136 patients who underwent pituitary adenoma resection. Compared with 188 patients who did not undergo dural suturing before 2005, it was confirmed that direct dural suturing in the sellar region could reduce dural defects and provide reliable support for cranial base structures. Even if watertight sutures cannot be achieved, this approach can effectively reduce the incidence of poCSF leakage; furthermore, approximately 72.92% (27/37) of the patients were exempted from autologous tissue transplantation. In 2015, Takeuchi et al. (24) reported a shoelace-type watertight suture method; the method consisted of using thin strips of autologous fat to fill the gap at the suture edge and continuously inserting needles at the edges of both sides of the dural incision at an interval of 2–3 mm to achieve watertight suturing. In 2018, the author (25) further enriched the concept of dural suturing and proposed a “three-level classification strategy”: for conventional transsphenoidal surgery, three-stitch conventional suturing can be performed. In the case of an extended transsphenoidal approach without obvious dural defects, shoelace watertight suturing is appropriate. In the case of an extended transsphenoidal approach with an obvious dural defect, an incision approximately 4–5 cm long must be made in the lower abdomen of the patient. The anterior rectus sheath and its surrounding adipose tissue should be taken as the material for graft repair, and then, the dura should be sutured with the autograft using the same shoelace watertight sutures mentioned above. In addition, Bederson et al. (26) introduced a special suture method for the dura mater on the sellar floor. They used a special titanium alloy U-clip to suture artificial dura mater or autologous fascia to the edge of the dura mater notch. The deployment time of a single U-clip was only 15–60 s. This method avoids the need to hold needles and knot sutures in the narrow nasal cavity. In 2019, Beijing Tiantan Hospital (27) shared experience with continuous suturing of the dura mater on the sellar floor in 36 patients with grade III intraoperative CSF leakage during pituitary adenoma surgery. Due to the large dural defect, the repair materials were obtained from autologous fascia. Compared with the control group (43 cases), the suture group showed a significantly reduced incidence of poCSF leakage [2.78% (1/36) vs. 20.93% (9/43), *p* = 0.016]. Surgeons at Jinling Hospital affiliated with the Medical College of Nanjing University performed (28) simple dural suturing without knotting using barbed sutures in 33 patients with intraoperative CSF leakage during transsphenoidal surgery. The time required was only 10 min, and difficult knotting operations were avoided. In our study, we attempted to reconstruct the cranial base by sellar dural suturing, and the results confirmed that patients indeed benefit from this technology. No patient in the suture group required preventive LD, and only one patient suffered poCSF leakage. The postoperative hospital stay was significantly shorter in the sutured group than in the control group, and the need for some invasive procedures such as harvesting NSF or autologous fat was reduced in the suturing group. The effect of sellar dural suturing is closely related to the operator's surgical experience and proficiency. There are some challenging aspects of dural suturing at the sellar region, such as the narrow operating space, the absence of stereopsis, and difficulty in knotting. When the research team first tried to perform dural suturing at the sellar region, each stitch required approximately 10 min and the whole suturing took 60 min, but the learning curve of this technique was short, and the suture duration could be stably controlled to within 20 min after approximately 20 operations. Excessive time consumption and technical challenges are major impediments to the popularity of sellar dural suturing. A too long reconstruction time is not recommended for EEA surgeries. We adopt a kind of 4–6 stitch interrupted suturing. Compared with watertight suturing, it is less time-consuming and less technically challenging. Our suture times are similar to those of scholars who have employed similar technology (Table 4). We believe that this operation is only technically challenging in the initial attempts and the suture time can be controlled for an acceptable duration after a certain amount of practices. From our experience, there are several points to note when performing a sellar dural suturing. First, when cutting the dura, it is necessary to reserve the suture position, ensuring that the bone window edge is at least 2 mm away from the dural window edge to facilitate needle entry. After incising the dura, electrocoagulation to the dura needs to be avoided to prevent dural contracture, which could increase the difficulty of suturing. For some lesions invading the dura, we need to remove the invaded dura and repair it with autologous fascia by suturing; finally, sellar dural suturing need to be completed in a relatively demanding bloodless surgical field. However, this suturing method may also have some limitations. For example, in cases of large dural defects, 4–6 intermittent sutures may not provide sufficient support for the sellar floor. For patients with severe dural tumor invasion, the dura may be too fragile, leading either to a poor effect or even the inability to suture.

**Table 4:** Time of dural suturing in endoscopic transsphenoidal surgery.

Reference	Suturing technique	Suturing time (min)		
		Initial attempt	When skilled	Average
Hai Xue et al. (27)	Watertight suturing	90	30–45	48
Jung Yong Ahn et al. (29)	Watertight suturing	560	50–90	NA
Takayuki Ishikawa et al. (25)	Watertight suturing	NA	30	NA
Hiroshi Nishioka et al. (23)	Intermittent suturing	NA	NA	30
Eui Hyun Kim et al. (30)	Intermittent suturing	NA	5–20	NA
Lijun Heng et al. (31)	Intermittent suturing	NA	5 per stitch	NA
Zixiang Cong et al. (28)	Barbed suturing	17	<10	10.33
Francesco Acerbi et al. (26)	Nitinol U-clips	1–10 per clip	0.25–1 per clip	NA
Current study	Intermittent suturing	60	11–26	20.56

### Limitations

The major limitations of this study are essentially the retrospective design and the limited number of patients. In addition, although we chose cases in the same period to avoid differences caused by the progression of surgical technology and operations performed without a senior expert were excluded to avoid differences caused by surgical experience, differences in surgeons’ preferences cannot be completely ruled out.

## Conclusion

Sufficient cranial base reconstruction is very important to avoid poCSF leakage. Sellar dural suturing is a new method with a perfect reparative effect. Although the initial operation is difficult, the learning curve is short. After professional training, most neurosurgeons can complete the suturing process within 20 min. Our results confirm that sellar dural suturing could effectively reduce the application of both autologous transplantation materials and LD and shorten the hospitalization duration. As a low-cost method for cranial base reconstruction, sellar dural suturing under endoscopy is promising and effective and is worthy of clinical application.

## Data Availability

The datasets presented in this study can be found in online repositories. The names of the repository/repositories and accession number(s) can be found in the article/Supplementary Material.
